# Soil fungal community and co-occurrence network patterns at different successional stages of black locust coppice stands

**DOI:** 10.3389/fmicb.2025.1528028

**Published:** 2025-03-18

**Authors:** Huimei Tian, Liangzhe Li, Yunpeng Zhu, Chengcheng Wang, Mengxue Wu, Weixing Shen, Chuanrong Li, Kun Li

**Affiliations:** ^1^Mountain Tai Forest Ecosystem Research Station of State Forestry Administration/Key Laboratory of State Forestry Administration for Silviculture of the Lower Yellow River/Forestry College of Shandong Agricultural University, Tai'an, China; ^2^Research Center for Forest Carbon Neutrality Engineering of Shandong Higher Education Institutions, Tai’an, China; ^3^Mount Tai Scenic Spot Management Committee, Tai'an, China

**Keywords:** black locust, stand conversion, fungal community, guild, co-occurrence network

## Abstract

**Background and aims:**

Black locust (*Robinia pseudoacacia* L.) plantations transition from seedling to multi-generation coppice systems, leading to declines in productivity and biodiversity. However, the structural and functional reorganization of soil fungal communities during this transition remains poorly understood. This study aimed to characterize fungal community dynamics across successional stages of black locust stands and assess their implications for soil health and ecosystem resilience.

**Methods:**

Soil fungal communities in three black locust stands (first-generation seedling forest, first- and second-generation coppice forests) were analyzed over one year using ITS high-throughput sequencing. We evaluated fungal diversity, guild composition, and co-occurrence networks, integrating statistical analyses (PERMANOVA, ANOSIM, FUNGuild) and network theory to assess seasonal and successional shifts.

**Results:**

Fungal richness and diversity remained stable across stand types and seasons. However, these factors dramatically altered the soil fungal community structure. Shifts in fungal community composition were observed from seedling to coppice stands: Ascomycota dominance decreased (72.9 to 57.9%), while Basidiomycota increased (6.5 to 11.6%). Significant changes in the relative abundance of certain fungal guilds were observed by both stand conversion and seasonal variation (*p* < 0.05). However, the overall fungal guilds composition was only significantly affected by the seasonal variation, rather than stand conversion (*p* > 0.05). Furthermore, saprotrophic fungi dominated in autumn/winter (66.49–76.01%), whereas symbiotic fungi peaked in spring (up to 7.27%). As forests transition from seeding to coppice stands, the percentage of negative edges, average degree, and relative modularity of the fungal community co-occurrence networks all gradually decreased. Those suggested that the conversion of black locust stands decreased the connectivity between fungal species, formed less organized structure, increased homogeneity of function among microbial communities, reduced ecological functionality, and decreased resistance to environmental changes. Seasonal temperature fluctuations further modulated network complexity, with summer samples showing heightened edge density but reduced cooperation.

**Conclusion:**

Our findings suggest that the conversion of forests can significantly shift the soil fungal community structure and assembly, favoring Basidiomycota over Ascomycota and reducing network stability. These shifts signal progressive soil nutrient depletion and functional homogenization, potentially compromising ecosystem resilience. Seasonal guild dynamics highlight fungi’s role in nutrient cycling, with saprotrophs driving litter decomposition in colder months. This understanding suggest that forest management practices must prioritise the preservation of early successional stages. This is vital to support diverse fungal communities and complex community networks and ensure the stability, functionality and resistance of fungal communities. Restoration efforts must focus on promoting fungal resilience through targeted soil amendments and habitat diversification to enhance ecosystem stability and functionality.

## 1 Introduction

Black locust (*Robinia pseudoacacia* L.), a nitrogen-fixing woody legume native to North America, has achieved global prominence due to its rapid growth, stress tolerance, and adaptability to degraded environments (Li et al., 2024; Seidel et al., 2024; Slabejová et al., 2023; Vítková et al., 2020). Introduced to China in the 19th century, this species now serves as a keystone component in ecological restoration programs, particularly for erosion control and soil amelioration in mountainous regions (Fan et al., 2023; Li et al., 2024; Xu et al., 2020). For instance, *R. pseudoacacia* was introduced to Mount Tai in the 1920s and afforestation activities were primarily conducted between 1956 and 1958 by seeding planting. Currently, extensive artificially planted black locust forests are concentrated on Mount Tai at elevations ranging from 500 to 1,000 meters on the southern slopes (Li et al., 2021; 2022). The reproduction of the black locust is achieved through both sexual and asexual methods. Despite its ecological benefits, recurrent harvesting-induced conversion from seedling stands to multi-generation coppice systems has triggered progressive declines in stand productivity and biodiversity, posing significant challenges to ecosystem sustainability (Šibíková et al., 2019; Su et al., 2023). Although prior studies have elucidated shifts in soil physicochemical properties and microbial biomass during this transition (Li et al., 2022; Seidel et al., 2024), critical knowledge gaps persist regarding the structural and functional reorganization of soil fungal communities-key mediators of biogeochemical cycles and plant-microbe symbioses (Baldrian, 2017; Frey, 2019).

Soil fungal communities are integral to forest ecosystems playing particularly pivotal roles in driving organic matter decomposition, nutrient cycling, and symbiotic interactions with plants (e.g., mycorrhizal networks) (Baldrian et al., 2023; Hagenbo et al., 2022; Zhou et al., 2023). The diversity of fungal communities serves as a vital indicator of ecosystem health. Rich and diverse fungal communities not only facilitate the evolutionary processes of plant communities but also enhance ecological functions critical for ecosystem restoration (Naka et al., 2024; Yang et al., 2024). Fungal communities orchestrate fundamental ecosystem processes through niche-specific functional guilds, which classify taxa based on their trophic strategies and ecological roles (Runnel et al., 2025). Modern metabarcoding approaches (e.g., ITS sequencing) coupled with functional databases like FUNGuild now enable precise guild characterization (Nguyen et al., 2016). Arbuscular mycorrhizal (AM) and ectomycorrhizal (EcM) fungi enhance plant mineral acquisition through root symbioses, while saprotrophs govern organic matter decomposition and carbon sequestration (Mundra et al., 2021; van der Heijden et al., 2015). In black locust plantations, guild-level analyses reveal that AMF dominance enhances plant phosphorus uptake in young stands, while saprotrophic fungi drive lignin degradation in mature forests, directly shaping soil carbon dynamics (Yang et al., 2022; Li et al., 2022). Shifts in guild composition during stand conversion, such as transitioning monocultures to mixed forests, can signal ecosystem recovery or degradation. A decline in EcM fungi, which mediate nitrogen transfer in mixed forests, may reduce tree growth resilience, whereas an increase in saprotrophs could accelerate litter turnover but destabilize soil organic carbon stocks (Marañón-Jiménez et al., 2021; Yang et al., 2024).

While traditional sequencing approaches have advanced our understanding of microbial diversity and composition, these methods often overlook the intricate web of interactions that govern community stability and function, such as cross-kingdom synergies, niche partitioning, and metabolic interdependencies. Network theory has emerged as a transformative framework to model these interactions, translating microbial co-occurrence patterns into interpretable topological structures (Deng et al., 2016; Jiao et al., 2017; Shi et al., 2016). By representing taxa as nodes and statistically robust associations as edges, networks reveal features like modularity (compartmentalized functional units), keystone taxa (disproportionately influential species), and cross-domain connectivity (e.g., bacterial-fungal partnerships) (Yang et al., 2022). Such networks provide insights into microbial community stability, niche partitioning, and functional redundancy. For instance, a higher proportion of negative interactions (competition) may indicate resource limitation, whereas positive interactions (cooperation) often reflect niche complementarity or shared environmental preferences (Zhou et al., 2023; Steidinger et al., 2019). Despite their ecological significance, few studies have integrated co-occurrence network analysis to assess how fungal communities respond to forest management practices, such as stand conversion, or how these responses vary seasonally (Babur et al., 2022; Voříšková et al., 2014).

Prior research on black locust forests has primarily focused on soil bacterial communities or arbuscular mycorrhizal fungi, overlooking broader fungal guilds and their functional shifts (Li et al., 2021; Sheng et al., 2017). For instance, Li et al. (2021) reported declines in soil nitrogen mineralization rates in coppice stands, while Sheng et al. (2017) linked changes in mycorrhizal fungal traits to soil nutrient dynamics. However, these studies did not address how fungal community composition, diversity, and interspecific interactions collectively adapt to stand conversion or seasonal fluctuations. Furthermore, while seasonal variations in microbial activity are well-documented in temperate forests (Kaiser et al., 2010; Voříšková et al., 2014), their impacts on fungal co-occurrence patterns-especially in monoculture plantations like black locust-remain unexplored. This knowledge gap limits our ability to predict the long-term ecological consequences of forest management strategies (Seidel et al., 2024; Wang et al., 2019).

The objectives of this study were to (1) characterize the structural and functional shifts in soil fungal communities across different successional stages of black locust stands (seedling, first-generation coppice, and second-generation coppice); (2) evaluate the stability and complexity of fungal co-occurrence networks in response to stand conversion; and (3) assess the seasonal dynamics of fungal guilds and their interactions. We hypothesized that H_1_: Fungal community composition and assembly would diverge significantly between seedling and coppice stands, with the greatest dissimilarity observed between seedling forests and second-generation coppice forests due to progressive soil nutrient depletion (Li et al., 2022; Zhan et al., 2021); H_2_: Coppice stands would exhibit simplified co-occurrence networks with reduced modularity and increased positive interactions, reflecting adaptive responses to harsher soil conditions (Deng et al., 2016; Egidi et al., 2019); H_3_: Seasonal changes would differentially influence fungal guilds across stands, with saprotrophic fungi dominating during litter-rich periods (autumn/winter) and symbiotic fungi peaking in active growth seasons (spring/summer) (Snajdr et al., 2011; Babur et al., 2021). By addressing these hypotheses, this study advances our understanding of how forest management practices reshape soil fungal ecology and provides actionable insights for optimizing black locust plantation strategies to enhance ecosystem resilience.

## 2 Materials and methods

### 2.1 Study area

The study sites are situated on Mount Tai in Shandong Province, China. This region features a northern temperate continental climate, with an average annual temperature of 12.8°C and mean annual precipitation of 1,124.6 mm. The existing stands of black locusts are predominantly coppice plantations, primarily located on south-facing slopes at elevations ranging from 500 to 1,000 meters. Three experimental sites were selected for the study, designated as follows: the first-generation sapling forest (referred to as “First” or “F,” coordinates: 36°16′45′′N, 117°3′26′′E); the first regeneration forest, formed by clearing the sapling forest (referred to as “Second” or “S,” coordinates: 36°16′40′N, 117°03′21′E); and the second-generation regeneration forest, obtained from clearing the first-generation regeneration forest (referred to as “Third” or “T,” coordinates: 36°16′40″N, 117°3′22″E). The dominant species within the understory vegetation community include *Vitex negundo*, *Oplismenus undulatifolius*, *Digitaria sanguinalis*, *Paspalum thunbergii*, *Rubia cordifolia*, and *Oxalis corniculate*. The soil type has been classified as Alfisols.

### 2.2 Soil sample collection

Soil sampling was conducted at three-month intervals from January 2019 to January 2020. In each seedling and coppice stand, a total of nine plots were established, with three plots randomly selected for sampling. In each season (winter, spring, summer, autumn), three random samples were taken from each plot, that is, nine samples were collected from each plot. Each plot measured 20 m × 20 m. Using a soil auger with a length of 50 cm, a diameter of 5 cm, and a volume of 100 cm^3^, bulk topsoil was collected from a depth of 10–15 cm in these nine plots using a shovel and thoroughly mixed. More details regarding the plots can be found in the paper by Li et al. (2021). Subsequently, the samples were transported on ice to the laboratory, where they were sieved through a 2 mm mesh size prior to being stored at −4°C for DNA extraction. In this manuscript, the acronyms FN, SN, and TN represent the bulk soils from the F, S, and T stands, respectively. The bulk soil samples from the five sampling periods were labeled T901, T904, T907, T910, and T001, respectively.

### 2.3 Soil microbial genome extraction

Total genomic DNA was extracted from 0.25 grams of fresh soil using a DNA Extraction Kit, following the manufacturer’s instructions (TIANamp Soil DNA Kit, TIANGEN). The concentration and quality of the extracted DNA were determined using a Nanodrop spectrophotometer (ND2000, Thermo Fisher Scientific, Waltham, MA, United States). The fungal Internal Transcribed Spacer 1 (ITS1) gene was amplified using the primer set ITS1F (5′-CTT GGT CAT TTA GAG GAA GTA A-3′) and ITS2R (5′-GCT GCG TTC TTC ATC GAT GC-3′). The polymerase chain reaction (PCR) products were purified using an AxyPrep DNA Gel Extraction Kit (Axygen Biosciences, Union City, CA, USA). The PCR amplification was performed in triplicate in a 50 μL reaction mixture, which consisted of 25 μL of 2 × PrimeSTAR Max Premix (Takara), 1.0 μL of each primer (10 μM), 20 ng of template DNA, with the remaining volume adjusted with double-distilled water. The PCR program included 30 cycles, comprising a denaturation step at 98°C for 10 s, an annealing step at 55°C for 15 s, and an elongation step at 72°C for 5 s. After amplification, the PCR products were assessed on a 2% agarose gel and subsequently purified using the AxyPrep DNA Gel Extraction Kit (Axygen Biosciences, Union City, CA, USA).

### 2.4 High throughput sequencing

The purified amplicons were meticulously prepared for high-throughput sequencing using the Illumina HiSeq2500 platform at Shanghai Biozeron Bio-pharm Technology Co., Ltd. (Shanghai, China), in accordance with rigorous standard operating procedures. After sequencing, the original DNA fragments were merged into pairs of reads using FLASH (Fast Length Adjustment of Short Reads) (Magoč and Salzberg, 2011). The raw sequences were demultiplexed and quality-filtered using the default parameters in the Quantitative Insights Into Microbial Ecology (QIIME) software package to obtain high-quality clean tags (Caporaso et al., 2010). The UCHIME algorithm was employed to detect chimeric sequences, which were subsequently removed (Edgar et al., 2011). The sequences were then classified into operational taxonomic units (OTUs) at 97% sequence identity using the UPARSE pipeline, and representative sequences for each OTU were selected (Edgar, 2013). The USEARCH_global tool was utilized to align all tags with their corresponding OTUs, generating a comprehensive statistics table for each sample (Edgar, 2010). The resulting representative sequence set was aligned and assigned with a taxonomic classification using the UNITE database at a minimal confidence threshold of 80% (Heděnec et al., 2020). Non-fungal sequences were excluded from subsequent analyses.

### 2.5 Data processing and statistical analysis

Prior to analysis, sequences were normalized based on the minimum size of each sample. The number of shared and unique OTUs among the groups was illustrated using UpSet Venn diagrams. Ternary plots were created to analyze the distributions of OTUs in the FN, SN, and TN groups. The alpha diversity of the fungal communities at the OTU level was calculated, and a Kruskal-Wallis test was applied to examine significant differences (*p* < 0.05) in richness, Shannon, ACE, and Simpson indices among the sampling groups. To evaluate beta diversity between different sample groups, principal coordinates analysis (PCoA), permutational multivariate analysis of variance (PERMANOVA), and analysis of similarities (ANOSIM) were employed, based on the Bray-Curtis distance between OTUs. A DeSeq analysis was conducted to assess the differences between species, with the following thresholds set: fold change >4 and *p* < 0.05, across three pairs (SN-FN, TN-FN and TN-SN). Additionally, the Statistical Analysis of Metagenomic Profiles (STAMP) software was utilized, employing the Kruskal-Wallis H-test method for the comparison of the three groups, while the Welch’s t-test method was used for pairwise comparisons at both the phylum and class levels (FDR-adjusted *p* < 0.05) (Parks et al., 2014). The ecological types of the fungal community were predicted using the FUNGuild analysis tool, which is based on the analysis of ITS sequences (Nguyen et al., 2016). In this study, we focused on the guilds identified by this tool. To reduce computational complexity and focus on the richest OTUs, we selected the 400 OTUs with the highest abundance (≥ 0.02%) in each sample for co-occurrence network analysis. OTUs with a Spearman correlation coefficient > 0.6 or < −0.6 and *p* < 0.05 were selected. The statistical analyses were performed in the R software (Version 4.3.0) and SPSS software (Version 27.0).

### 2.6 GenBank submission and accession numbers

The high-throughput sequencing-derived ITS gene sequences have been deposited in the National Center for Biotechnology Information (NCBI) Sequence Read Archive (BioProject ID: PRJNA1159511).

## 3 Results

### 3.1 OTU distribution of fungal communities across samples

**Figure 1:**
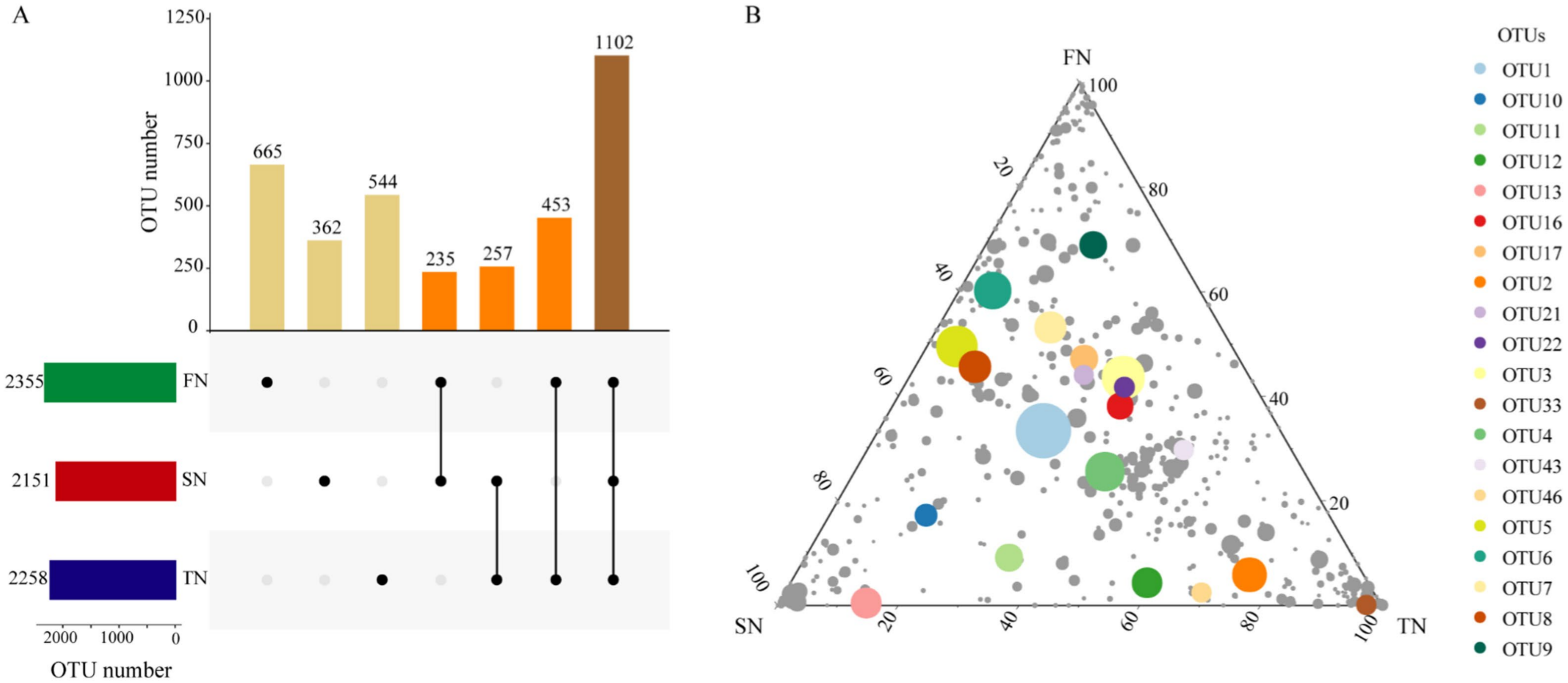
OTU distribution of fungal communities in the FN, SN and TN groups. **(A)** UpSet Venn diagram showing the shared and unique OTUs in different sample groups. **(B)** Ternary plots of OTUs detected in three groups. Only OTUs found in at least 1‰ of the samples are included in the figure and each symbol represents a single OTU. The top 20 OTUs with high relative abundance are labeled with different colors. The size and position of each symbol represents its relative abundance and affiliation of the OTU with different groups.

Amplicon sequencing of the ITS was conducted to determine the composition of the fungal community. A total of 225,576, 218,054, and 200,195 sequences were obtained after merging and filtering the raw data of ITS tags, yielding an average of 45,115, 43,610 and 40,039 sequences per sample for the FN, SN and TN groups, respectively. The coverage of the samples ranged from 98.7 to 99.3%, indicating that the sequencing effort was sufficient to describe the vast majority of fungal communities present in the samples. Totally, 2,355, 2,151 and 2,258 OTUs were obtained for the FN, SN and TN groups, respectively. An UpSet Venn diagram was employed to illustrate the shared OTUs among the three sample groups (Figure 1A). The proportion of shared OTUs was 46.8, 51.2, and 48.8%, while the unique OTUs constituted 28.2, 16.8, and 24.0% of the total OTUs in the FN, SN, and TN groups, respectively. Notably, the FN and TN groups exhibited the highest degree of overlap in terms of OTUs, whereas the FN and SN groups displayed the lowest level of shared OTUs. The distribution of OTUs was illustrated using ternary plots (Figure 1B), which revealed significant differences in the top 20 OTUs with high relative abundance among the three groups. Specifically, the FN group exhibited the highest relative abundance of different numbers of top OTUs (10 OTUs), SN group (4 OTUs), and TN group (6 OTUs). Additionally, the FN group exhibited lower proportions (<10%) of six specific OTUs, including OTU 2, OTU 33, OTU 46, OTU 12, OTU 11, and OTU 13, representing less than 10% of the total. It is noteworthy that OTU 33 demonstrated relatively low distribution within the SN group, while three specific OTUs (OTU 5, OTU 8, OTU 6) exhibited lower proportions within the TN group.

### 3.2 Diversity of fungal communities across samples

**Figure 2:**
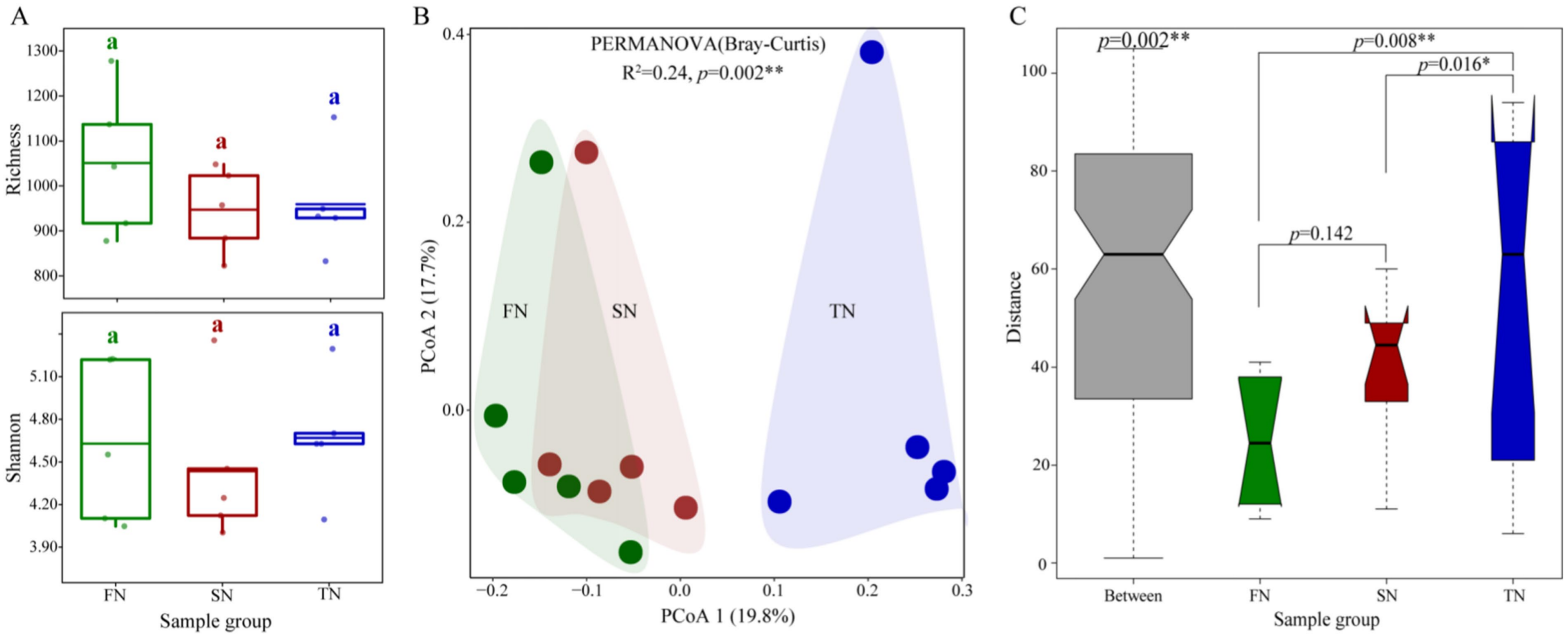
Diversities of fungal communities in the FN, SN and TN groups. **(A)** Comparison of Richness and Simpson indexes of fungal communities. Horizontal bars in the box plots indicate median proportional values. Kruskal-Wallis test was applied to examine differences and different lower-case letters indicate significant differences (*p* < 0.05) among sampling groups. **(B)** The principal coordinate analysis (PCoA) and PERMANOVA analysis of the fungal communities in the three different sample groups based on Bray-Curtis distance at the OTU level. **(C)** Anosim analysis of the fungal communities in the three different sample groups based on Bray-Curtis distance at the OTU level.

The species richness and Chao 1 values for the FN group (mean = 1,051 and 1,392, respectively) were found to be higher than those for the SN group (mean = 947 and 1,194, respectively) and the TN group (mean = 959 and 1,213, respectively). However, the observed differences were not statistically significant (*p* > 0.05). Regarding the Shannon diversity index and the Shannon evenness index, the FN group (mean = 4.629 and 0.963, respectively) and the TN group (mean = 4.669 and 0.969, respectively) exhibited comparable values, both of which were higher than those observed in the SN group (mean = 4.436 and 0.956, respectively) (Figure 2A; Supplementary Figure S1A; Supplementary Table S1).

According to the Bray-Curtis distance among OTUs, principal coordinates analysis (PCoA), permutational multivariate analysis of variance (PERMANOVA), and analysis of similarities (ANOSIM) were employed to assess *β* diversity among different sample groups (Figures 2B,C). The PCoA plot showed that the first two axes accounted for 37.5% of the variation in the fungal community. The fungal communities in the FN and SN groups were observed to cluster together while exhibiting a clear separation from those in the TN group. The PERMANOVA analysis yielded statistically significant results, confirming a notable separation among the fungal communities in the three sample groups (*p* = 0.002). A similar outcome was evident in the ANOSIM results, which indicated that the divergence in fungal communities across the FN, SN, and TN groups was statistically significant (*p* = 0.002, Figure 2C). The results of the ANOSIM pairwise comparison revealed significant differences between the fungal communities in the TN group and those in the FN and SN groups (*p* = 0.008 and 0.016, respectively). However, no significant difference was observed between the FN and SN communities (*p* = 0.142). A partial least squares discriminant analysis (PLS-DA) was also conducted to identify differences among the three groups. The results demonstrated that the fungal communities of the FN, SN and TN groups were partitioned into three distinct quadrants, with the TN group being segregated from the other two groups in the first dimension (Supplementary Figure S1B). Over time, no significant differences were observed in the *α* diversity index of samples collected at different sampling times. In terms of fungal community structure, the ANOSIM analysis revealed significant overall differences; however, no significant differences were found in pairwise comparisons (Supplementary Table S2).

### 3.3 Assembly pattern of fungal community across samples

**Figure 3:**
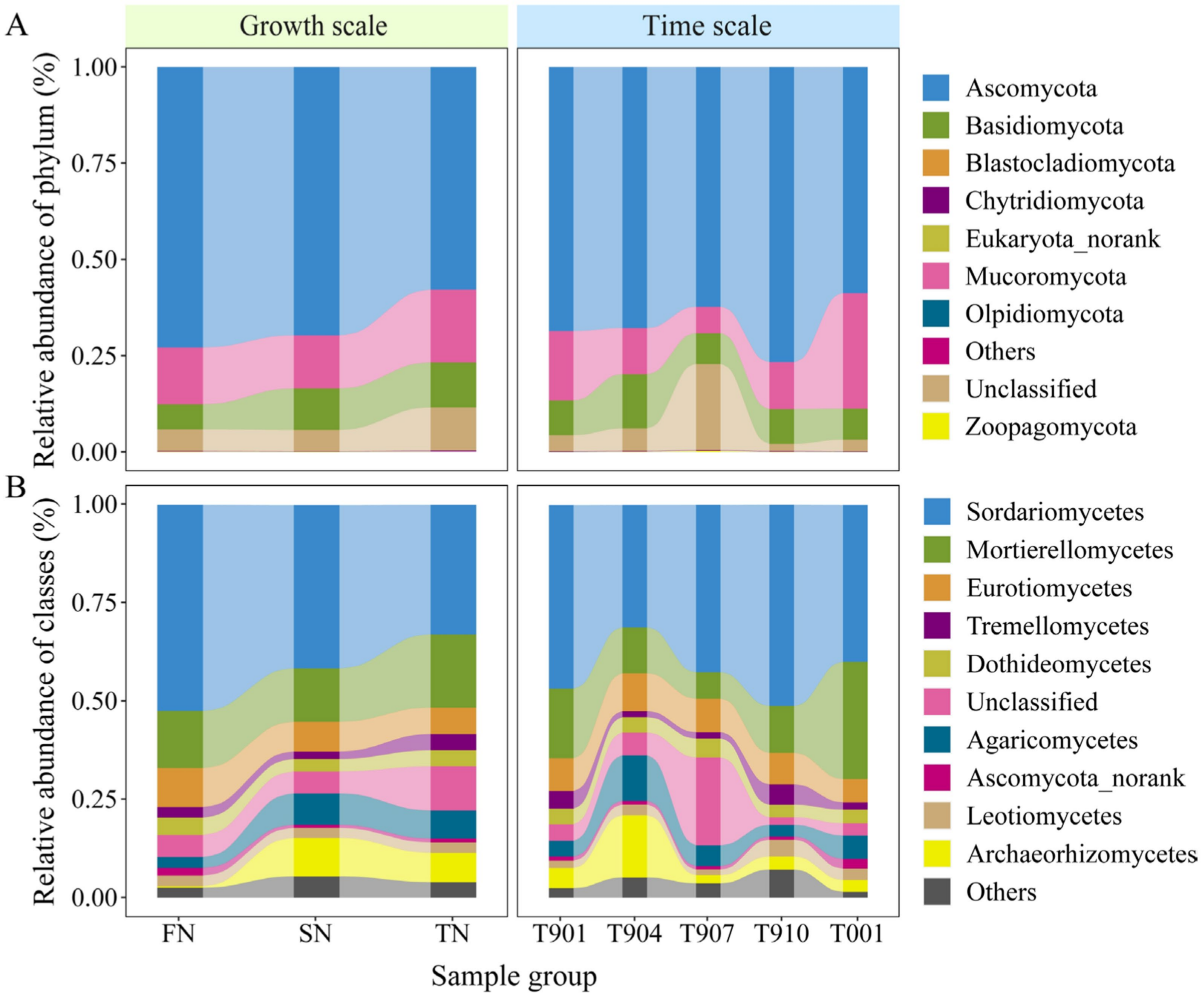
Distribution of fungal communities of each sample at the phylum level **(A)** and the class level **(B)**. Class-level fungal community composition only showed the top 10 classes with the highest relative abundance in the bar plots.

Shifts in fungal community composition were observed from the FN group to the SN and TN groups (Figure 3). In total, the reads were classified and grouped into eight defined phylum-level taxonomic categories: Ascomycota, Basidiomycota, Blastocladiomycota, Chytridiomycota, Eukaryota_norank, Mucoromycota, Olpidiomycota, and Zoopagomycota. In the FN, SN and TN groups, the most prevalent fungal phylum was Ascomycota, representing an average of 72.9, 69.7, and 57.9% of the fungal communities, respectively. This was followed by Mucoromycota, which constituted an average of 14.8, 13.8, and 18.9% of the fungal communities, respectively. Basidiomycota also made a notable contribution, representing an average of 6.5, 10.8 and 11.6% of the fungal communities, respectively. The unclassified phyla accounted for approximately 5.6, 5.6, and 11.3% of the total fungal communities in the FN, SN and TN groups, respectively. The relative abundance of the remaining four phyla in all samples was negligible, measuring less than 0.1% (Figure 3A). At the class level, a total of 42 defined taxonomic groups were identified. Only the ten most abundant classes were plotted, revealing significant differences in the fungal microbial compositions among the three groups (Figure 3B). Across all samples, the most prevalent fungal groups were Sordariomycetes and Mortierellomycetes, with relative abundances reaching up to 52.5 and 14.6%, 41.5 and 13.6%, and 33.0 and 18.7% in the FN, SN, and TN groups, respectively. Eurotiomycetes also exhibited high relative abundance in the FN group, while the relative abundances of Archaeorhizomycetes and Agaricomycetes were elevated in the SN and TN groups. Furthermore, the temporal changes in fungal community structure at both the phylum and class levels were consistent across the three groups, with samples marked as T907 exhibiting significant dissimilarity from the other samples (Supplementary Figure S2). The PLS-DA analysis provides a more comprehensive and visually representative depiction of the temporal succession process of fungal communities, which tend to maintain their initial state as time progresses (Supplementary Figure S1C).

**Figure 4:**
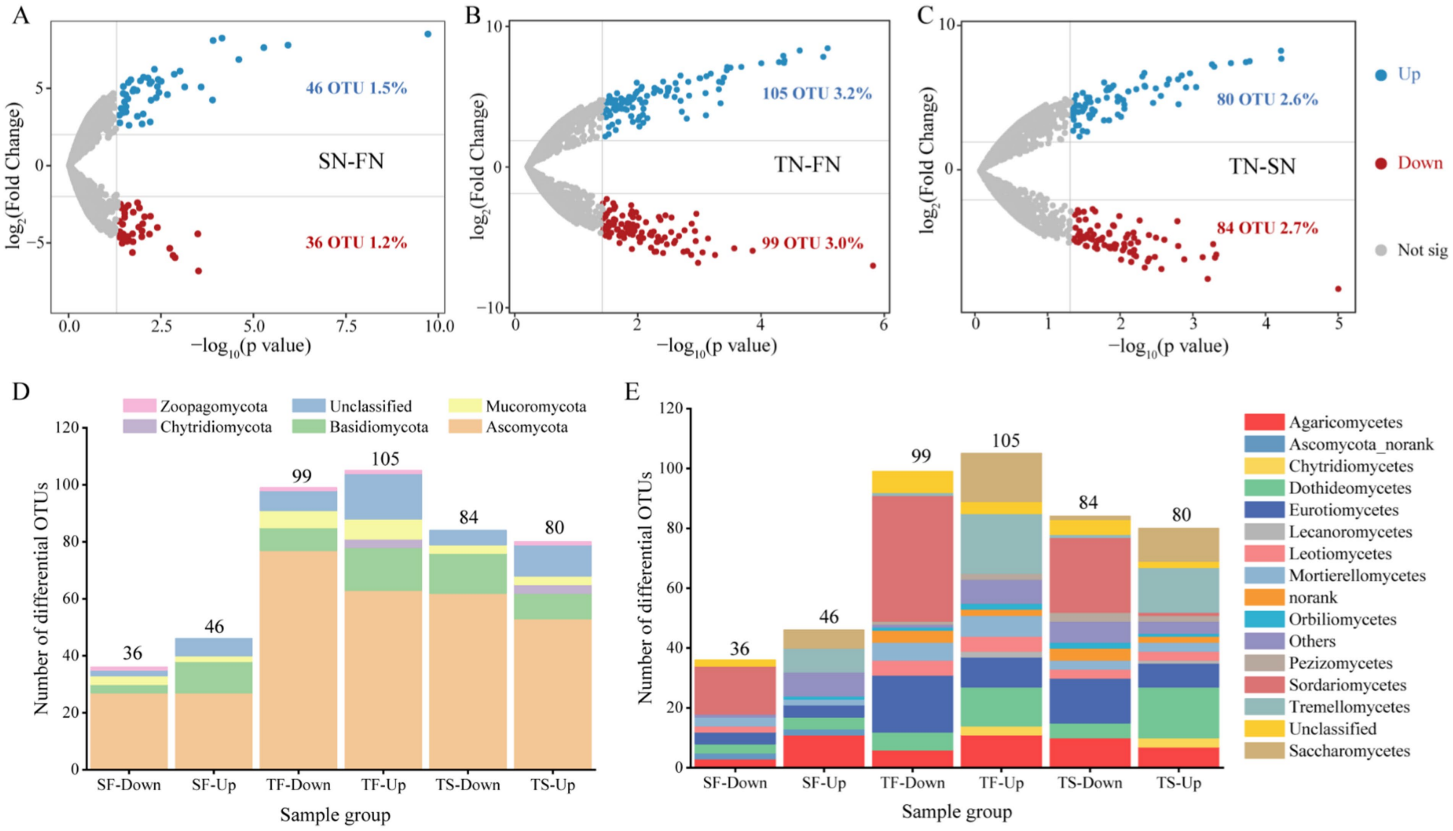
Differential community composition. **(A–C)** Volcano plots of OTUs detected in the FN, SN and TN groups. Each symbol represents a single OTU. The DeSeq analysis was conducted and the thresholds set at fold change >4 and *p* < 0.05. Blue and red depicts OTU significantly enriched and decreased in treated samples compared with the control, respectively. Grey symbols represent OTUs without significant differences. **(D–E)** The taxonomic classification and composition of differential OTUs at the phylum and class levels. SF represents a comparison between the SN and FN groups, TF represents a comparison between the TN and FN groups, and TS represents a comparison between the TN and SN groups.

The distinct OTUs between the sample groups were identified using the volcano plots, as illustrated in Figure 4. A comparison of the FN and SN sample groups revealed a significant enrichment of 46 OTUs and a significant decrease of 36 OTUs in the SN group (Figure 4A). In contrast to the FN group, the TN sample group exhibited a considerably higher number of enriched and decreased OTUs, totaling 105 and 99, respectively (Figure 4B). A comparison between the TN and SN groups revealed that 80 OTUs were significantly enriched, while 84 OTUs were significantly decreased (Figure 4C). At the phylum level, the majority of the upregulated and downregulated OTUs were affiliated with Ascomycota and Basidiomycota across all three comparison pairs (Figure 4D). A notable distinction was observed at the class level in the community composition of the upregulated and downregulated OTUs, with Agaricomycetes and Sordariomycetes representing the predominant upregulated OTUs in the SN-FN group. In contrast, Sordariomycetes and Dothideomycetes were predominantly observed as upregulated OTUs in both the TN-FN and TN-SN groups. Regarding the downregulated OTUs, Sordariomycetes were the most prevalent in the SN-FN group, while Sordariomycetes and Eurotiomycetes were the most commonly identified in both the TN-FN and TN-SN groups.

At the phylum level, only the relative abundance of Ascomycota showed statistical differences (*p* < 0.05) among the three groups (Table 1). At the class level, five species demonstrated statistically significant differences, namely Sordariomycetes, Archaeorhizomycetes, Chytridiomycetes, Lichinomycetes, and Pucciniomycetes. In pairwise comparisons within the FN-TN group, only Ascomycota (*p* < 0.01) and Basidiomycota (*p* < 0.05) showed significant differences. At the class level, the FN-TN and SN-TN comparison groups exhibited distinct differences in the abundance of Sordariomycetes, Lichinomycetes and Chytridiomycetes. A comparison of each group with the other two groups revealed that Basidiomycota differed significantly in all comparisons at the phylum level. Additionally, Ascomycota exhibited significant differences (*p* < 0.01) when comparing the TN with the other two groups. At the class level, the TN group had the highest number of distinct species, followed by the FN group when compared to the two other groups. The identified distinct species belonged to six classes in the TN group (Sordariomyces, Classiculomyces, Lichinomyces, Atractiellomyces, Monoblepharidomyces, Blastocladiomyces), four classes in the FN group (Sordariomycetes, Archaeorhizmycete, Lichinmycete, Agaricomcytes), and three classes in the SN group (Chytridimcytes, Lichnmycte, Zoopagmcyta_norank). On the time scale, differential species were observed in pairs at both phylum and class levels (Supplementary Table S3). Mucoromycota and Zoopagomycota differed in five and four pairs, respectively. Among the differential classes, the Mortierellomycetes differed in five pairs.

**Table 1:** Difference analysis of fungi species across FN, SN and TN groups at phylum and class level by STAMP at 95% confidence interval.

Fungi	FN vs. SN vs. TN^a^	FN vs. SN^b^	FN vs. TN^b^	SN vs. TN^b^	FN vs. Others^b^	SN vs. Others^b^	TN vs. Others^b^
Ascomycota	*	ns	**	ns	ns	ns	**
Basidiomycota	ns	ns	*	ns	*	ns	*
Chytridiomycota	ns	ns	ns	ns	ns	*	ns
Number of differential phylum	1	0	2	0	1	1	2
Sordariomycetes	*	ns	*	ns	*	ns	**
Archaeorhizomycetes	**	ns	ns	ns	*	ns	ns
Chytridiomycetes	*	ns	ns	*	ns	*	ns
Lichinomycetes	*	ns	*	*	*	*	*
Pucciniomycetes	*	ns	ns	ns	ns	ns	ns
Agaricomycetes	ns	ns	ns	ns	*	ns	ns
Zoopagomycota_norank	ns	ns	ns	ns	ns	*	ns
Classiculomycetes	ns	ns	ns	ns	ns	ns	**
Atractiellomycetes	ns	ns	ns	ns	ns	ns	*
Monoblepharidomycetes	ns	ns	ns	ns	ns	ns	*
Blastocladiomycetes	ns	ns	ns	ns	ns	ns	*
Number of differential classes	5	0	2	2	4	3	6

a Krusksl-Wsllis H-test.

b Welch’s *t*-test.

* represents 0.01 < *p* < 0.05; ** represents 0.001 < *p* < 0.01; *** represents *p* < 0.0001; ns represents not significant.

### 3.4 Fungal community guilds across samples

**Figure 5:**
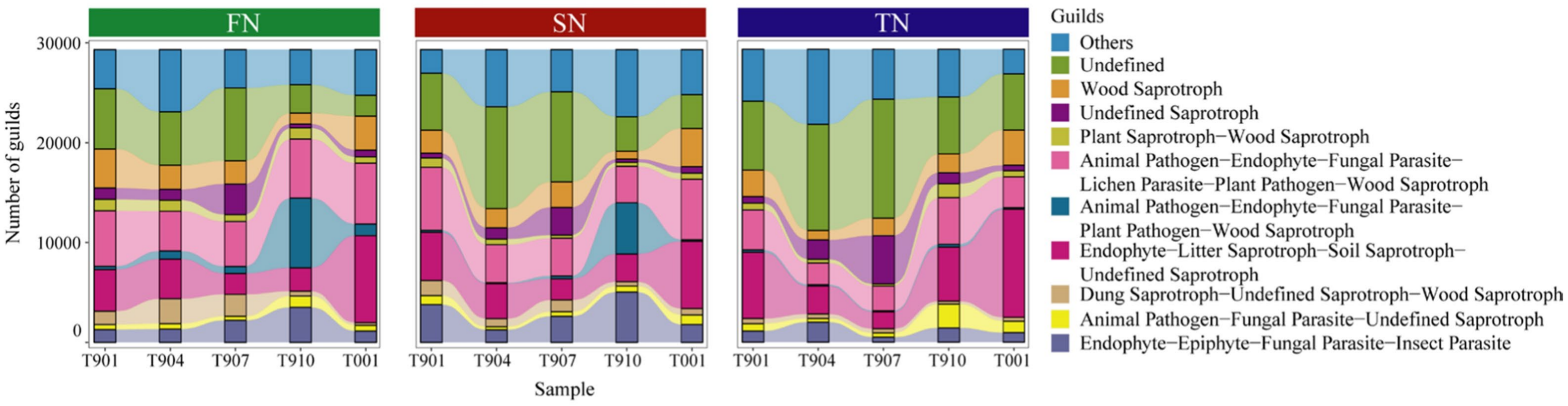
Distribution of fungal community guilds across samples in the FN, SN and TN groups. Only the top 10 guilds with the highest abundance were shown in the bar plots.

Here, the fungal community guilds of samples were predicted using FUNGuild, identifying a total of 101 guilds. Among these, Undefined Saprotroph, Animal Pathogen-Endophyte-Fungal Parasite-Lichen Parasite-Plant Pathogen-Wood Saprotroph Endophyte-Litter Saprotroph-Soil Saprotroph-Undefined Saprotroph and Endophyte-Epiphyte-Fungal Parasite-Insect Parasite were found in high proportions (Figure 5). Statistical analysis revealed no significant differences in the composition of fungal community guilds among the FN, SN and TN sample groups (*p* > 0.05) (Supplementary Figure S3A). However, difference analysis identified six guilds that varied the three sample groups: Algal Parasite-Bryophyte Parasite-Fungal Parasite-Undefined Saprotroph, Animal Pathogen-Endophyte-Fungal Parasite-Plant Pathogen-Wood Saprotroph, Animal Pathogen-Plant Pathogen-Undefined Saprotroph, Ectomycorrhizal-Fungal Parasite-Soil Saprotroph-Undefined Saprotroph, Fungal Parasite and Plant Pathogen-Undefined Saprotroph (Table 2). Comparative analysis between the two sample groups showed that the FN and TN sample groups had the highest number of distinct guilds (four guilds), followed by the FN and SN sample groups (two guilds), while the SN and TN sample groups displayed the lowest number of different guilds (one guild).

**Table 2:** Guilds with significant difference across soil sample groups by STAMP at 95% confidence interval.

Guilds	FN vs. SN vs. TN^a^	FN vs. SN^b^	FN vs. TN^b^	SN vs. TN^b^
Algal parasite-bryophyte parasite-fungal parasite-undefined saprotroph	**	ns	ns	ns
Animal pathogen-endophyte-fungal parasite-plant pathogen-wood saprotroph	*	ns	ns	ns
Animal pathogen-plant pathogen-undefined saprotroph	*	ns	ns	ns
Ectomycorrhizal-fungal parasite-soil saprotroph-undefined saprotroph	*	ns	ns	ns
Fungal parasite	*	ns	ns	ns
Plant pathogen-undefined saprotroph	*	ns	ns	ns
Plant saprotroph-wood saprotroph	ns	*	ns	ns
Animal pathogen-plant pathogen-undefined saprotroph	ns	*	ns	ns
Fungal parasite	ns	ns	**	ns
Animal pathogen-plant pathogen-undefined saprotroph	ns	ns	*	ns
Lichenized	ns	ns	*	*
Animal pathogen-endophyte-fungal parasite-lichen parasite-plant pathogen-wood saprotroph	ns	ns	*	ns
Number of differential guilds	6	2	4	1

a Krusksl-Wsllis H-test.

b Welch’s *t*-test.

* represents 0.01 < *p* < 0.05; ** represents 0.001 < *p* < 0.01; *** represents *p* < 0.0001; ns represents not significant.

Over time, ANOSIM analysis revealed significant differences in the fungal community guilds across five groups at different sampling times (*p* = 0.001) (Supplementary Figure S3B), but no significant differences were found between the two sample groups. Regarding differential guilds, five guilds varied across all sample groups: Endomycorrhizal-Plant Pathogen-Undefined Saprotroph, Endophyte-Litter Saprotroph-Soil Saprotroph-Undefined Saprotroph, Undefined Saprotroph, Wood Saprotroph, Endophyte-Lichen Parasite-Plant Pathogen-Undefined Saprotroph, as well as Fungal Parasite-Plant Pathogen-Plant Saprotroph. Intergroup variation in fungal community guild composition and differential guild was observed, with 1 (T904-T910) to 7 (T907-T001) differential guilds for two comparison groups (Supplementary Table S4). Additionally, we specifically analyzed two fungal guilds—saprophytic and symbiotic fungi—and observed distinct distribution patterns (Supplementary Table S5). The average proportion of saprophytic fungi in FN samples (71.14%) was approximately 10% higher than in SN and TN samples (61.27 and 61.39%), whereas symbiotic fungi were more abundant in SN and TN samples than in FN samples. Over time, these differences became more pronounced. Saprophytic fungi predominantly occurred in samples collected during autumn and winter (66.49–76.01%), including T901, T910, and T001, while symbiotic fungi peaked in spring, exemplified by T904 (7.27%), with other time periods ranging from 1.27 to 2.23%.

### 3.5 Fungal community networks

**Figure 6:**
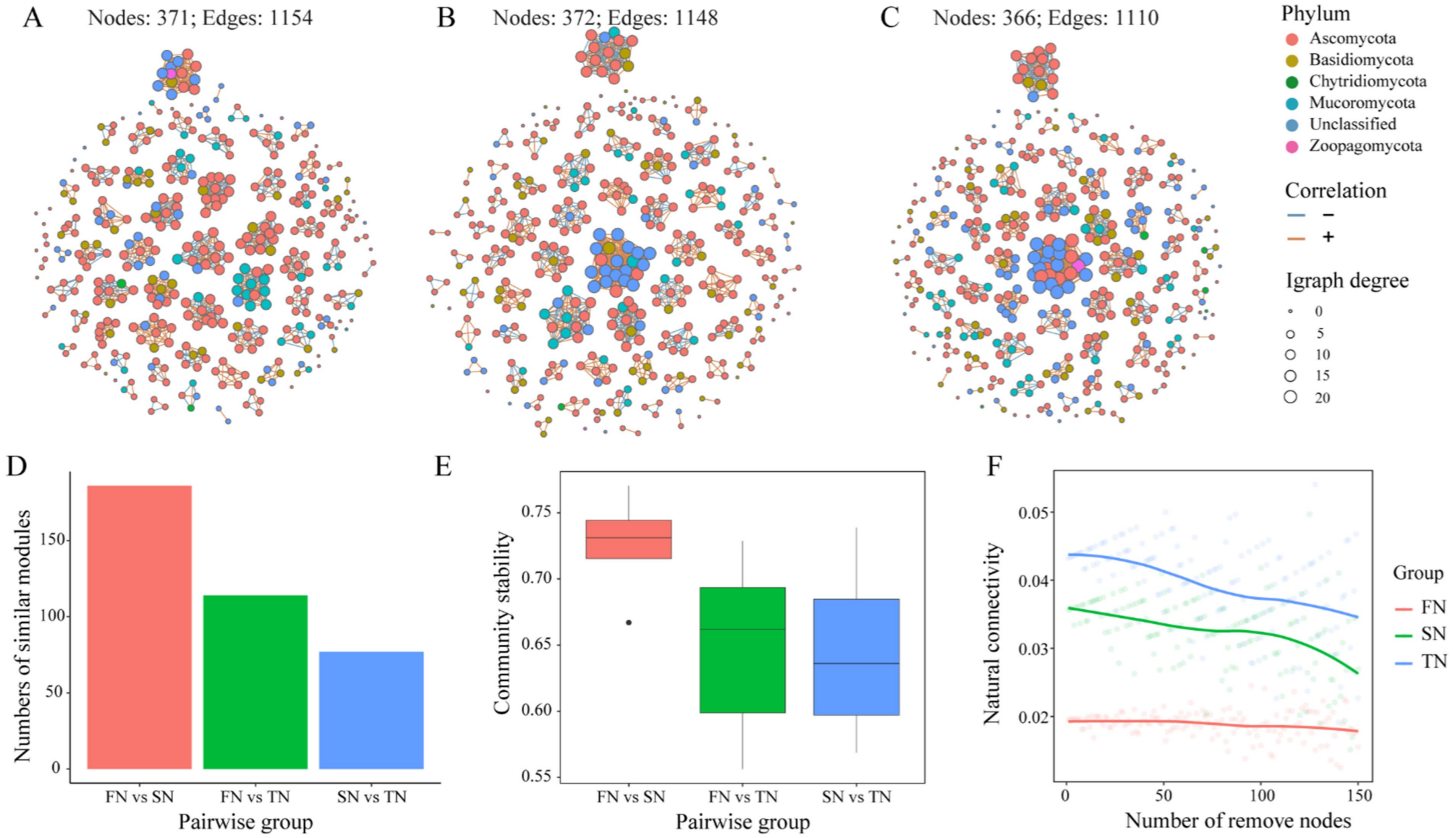
Co-occurrence network of the 400 most abundant OTUs in the FN, SN and TN samples **(A–C)**. A connection stands for OTUs with Spearman correlation coefficient > 0.6 or < −0.6 and *p* < 0.05 were determined. The size of each node is proportional to the degree. The red edges indicate positive interactions between two OTUs, while blue edges indicate negative interactions. Colors of nodes in network represent the bacterial phyla. Characteristics of co-occurrence networks of soil fungal communities **(D–F)**. **(D)** Comparison of numbers of similar modules in pairs. **(E)** Comparison of community stability in pairs. **(F)** Natural connectivity of co-occurrence network.

The co-occurrence networks were constructed based on Spearman’s coefficients and differences in the structures and topological properties of the networks were found among the three sample groups. Differences in the phylum composition of the modules were observed between the groups (Figures 6A–C). Ascomycota and Basidiomycota dominated the network of the FN group, whereas Ascomycota, Basidiomycota, and Unclassified dominated the networks of the SN and TN groups. Compared to the FN group, both the networks of TN and SN groups exhibited a decrease in the significance of Ascomycota, while there was an increase in the importance of Unclassified taxa. In addition, we observed that the TN and SN groups have developed a more prominent fungal flora consisting mainly of Unclassified species, which was further supported by a higher centralisation degree than the FN group. Topologically, there are obvious regularities between the sample groups based on their growth scale (Table 3). In the order of FN, SN, and TN, the percentage of positive edges (0.655, 0.692 and 0.717), the number of clusters (67, 73 and 75), the centralisation degree (0.018, 0.037 and 0.046), and the random modularity (0.311, 0.325 and 0.361) in the whole network gradually increased, while the proportion of negative edges (0.345, 0.308 and 0.283), the average degree (6.221, 6.172 and 6.066), and the relative modularity (2.097, 1.894 and 1.542) in the whole network gradually decreased. The comparison of modules across different groups shows that FN and SN have the highest number of similar modules, followed by FN and TN, while SN and TN have the lowest level of module overlap (Figure 6D). Thus, both SN and TN groups generated relatively less complex network patterns compared to the FN group. Comparison and analysis of community stability between paired groups revealed that the difference in community composition was most pronounced between FN and SN groups, followed by FN and TN groups, while the difference between SN and TN groups was less pronounced (Figure 6E). Regarding the resilience of the fungal networks (Figure 6F), the natural connectivity of the FN group exhibited a slow decline with node removal, indicating that even if some microbial species were lost, the network could still maintain robust connectivity. This observation reflects the strong stability of the microbial community structure. Conversely, both SN and TN groups experienced a rapid and significant decrease in natural connectivity with node removal, suggesting a lower resistance to perturbation within their respective communities.

**Table 3:** Topological properties of co-occurrence networks for OTU composition of soil samples across different groups.

Properties	FN	SN	TN
Number of nodes	371	372	366
Number of edges	1,154	1,148	1,110
Percentage of positive edges	0.655	0.692	0.717
Percentage of negative edges	0.345	0.308	0.283
Edge density	0.017	0.017	0.017
Average degree	6.221	6.172	6.066
Number of clusters	67	73	75
Centralization degree	0.018	0.037	0.046
Relative modularity	2.097	1.894	1.542
Modularity random	0.311	0.325	0.361

On the time scale, obvious differences in both the structures and the topological properties of the networks were detected (Supplementary Figure S4; Supplementary Table S6). Ascomycota and Basidiomycota were the predominant phyla in the networks of T901, T904, and T910, while Unclassified was notably present in the networks of T907 and the proportion of Mucoromycota increased in the networks of T001. In terms of topological properties, the network of T907 showed significant differences compared to the other networks, including having the highest number of edges, the lowest percentage of positive edges (24772), the highest edge density (0.310), and highest average degree (123.86). The network of T901 had the highest relative modularity (17.106) and the lowest centralisation degree (0.057); the network of T904 had the highest percentage of positive edges (0.618) and the highest centralisation degree (0.168); while the network of T910 had the highest random modularity (0.046) and the lowest average degree (99.56). The topological properties of network T001 were very similar to those of group T901. Group T904 showed lower resilience to fungal network disruption within their respective communities as their natural connectivity decreased with node removal, in contrast to the remaining four groups whose natural connectivity remained relatively stable despite node removal (Supplementary Figure S5).

## 4 Discussion

In this study, we used high-throughput sequencing to analyze the composition, guild, and co-occurrence network patterns of soil fungal communities in three black locust stands over the course of one year. Based on our previous study, where significant shifts in soil diazotrophs were observed between the seedling and coppice plantations, we expected similar changes in the fungal community, as the soil properties showed obvious differences, since after two or three rotations and continuous generations of black locust, the productivity of black locust coppice plantations tends to decline (Li et al., 2021, 2022; Šibíková et al., 2019). Indeed, significant differences in soil fungal community composition were observed among the three black locust plantations, especially between the FN (planted seedling stand) and TN (second coppice stand) groups (Figure 2), which was consistent with our first research hypothesis. Those results suggested that the effects of forest conversion on fungal community structure may be progressive. It has been speculated that this may be due to the diverse growth characteristics of black locust stands and their soil conditions (Sheng et al., 2017), specially soil available nitrogen (Li et al., 2021, 2022). Representing a critical component of the forest soil microbial community, the composition and dynamics of the fungal community can serve as potential indicators for assessing the soil properties and growth status of *R. pseudoacacia* forests. Modulating the structure of the fungal community may improve the growth conditions of these forests, which is essential for effective forest management and ecological restoration (Fan et al., 2023; Li et al., 2024; Sheng et al., 2017), especially from the perspective of microbe-plant interactions. Unlike the fungal community composition, although the richness and diversity of fungal communities were similar across all stands, the FN group had the highest richness, while the TN group had the highest diversity (Figure 2). This could be attributed to the complex regulation of fungi in response to various environmental factors, including temperature, humidity, pH levels, and other conditions.

Fungal community dynamics in forest ecosystems exhibit distinct functional stratification between Basidiomycota and Ascomycota phyla. Studies demonstrate that Basidiomycota dominates in forest soils with high organic matter content, while Ascomycota-characterized as copiotrophic organisms with rapid growth strategies, plays vital roles in soil stabilization, plant biomass decomposition, and endophytic symbiosis within arid grassland ecosystems (Challacombe et al., 2019; Su et al., 2023; Zhan et al., 2021). Notably, the decline of Ascomycota populations serves as a bioindicator for deteriorating soil quality (Li et al., 2021, 2022; Zhan et al., 2021). Comparative analysis between forest management groups revealed significant ecological shifts: SN and TN groups showed reduced Ascomycota dominance alongside increased Basidiomycota abundance compared to FN controls (Figure 3). This competitive displacement aligns with their ecological strategies, dominant species typically exhibit broad niche occupation and superior resource utilization efficiency (Jiao et al., 2017). Particularly during organic matter decomposition processes, researchers observed a consistent transition from Ascomycota to Basidiomycota predominance (Wang et al., 2019). This successional pattern likely stems from substrate availability changes, where Basidiomycota’s enhanced capacity for complex compound decomposition becomes advantageous as labile substrates diminish (Wang et al., 2019). The functional dichotomy between these phyla profoundly impacts ecosystem processes. Basidiomycota’s lignocellulose degradation capabilities contrast with Ascomycota’s preference for simpler compounds, collectively driving nutrient cycling dynamics that ultimately regulate plant growth parameters. These findings underscore the critical role of microbial community structure in maintaining forest ecosystem productivity, with dominant fungal taxa acting as key mediators of plant–soil interactions.

Fungal saprotrophs are known to strongly influence decomposition processes, decomposing litter and converting organic matter into plant-available nutrients including inorganic nitrogen (Phillips et al., 2013; Steidinger et al., 2019). Here, most soil fungi were classified as saprotrophic fungal guild ranging from 47.41 to 79.82% (Supplementary Table S5), which is consistent with previous research showing that *R. pseudoacacia* forest soils are dominated by fungal saprotrophs rather than ectomycorrhizal fungi (Seidel et al., 2024). Comparative analysis did not reveal any significant differences in the guild profiles of the soil fungal communities within the three black locust stands, but different guilds were detected (Supplementary Figure S3; Table 2). Furthermore, we observed that the number of differential guilds was most pronounced between FN and SN plantations, particularly with regard to parasitic and pathogenic fungi, such as Fungal Parasite and Algal Parasite-Bryophyte Parasite-Fungal Parasite-Undefined Saprotroph. Thus, as the black locust forest was converted, there was a gradual decrease in harmful soil fungal groups, indicating that the coppice stand was less susceptible to fungal infections. Another notable finding was the presence of different fungal guilds associated with symbiosis in the TN group, for example, Ectomycorrhizal and Ectomycorrhizal-Fungal Parasite-Soil Saprotroph-Undefined Saprotroph. This may be related to the different nutrient uptake strategies of the seedling stand and the second coppice stand, as the trees’ access to nitrogen bound in organic forms is tightly dependent on ectomycorrhizal fungi (Smith and Read, 2008). Changes in ecological types reflect the transformation and optimization of their functions in the ecosystem, as well as the composition and structural changes in forest soil fungal communities. These functional changes are of great importance for the health, stability and sustainable development of forest ecosystems. Furthermore, the guild of many fungal species in black locust stands was previously unknown, and their relative abundance increased with the forest conversion (up to 12.7%) (Undefined guild in FN was 16.1%, while in TN was 28.8%), emphasizing the importance of gaining a more comprehensive understanding of the fungal communities inhabiting this species, as they may be associated with the decline of black locust.

Network analysis is well suited to assess the collective abundance of microorganisms and to gain a thorough comprehension of microbial community structure and assembly patterns (Deng et al., 2012; Faust and Raes, 2012). Consistent with our second research hypothesis, as forests transition from seeding to coppice stands, the percentage of negative edges, average degree, and relative modularity within fungal community co-occurrence networks all gradually decrease in the order of FN, SN, and TN. Specially, the decrease in the percentage of negative edges signified a greater prevalence of synergistic interactions among microbial species within the network. This trend likely reflects a cooperative response to environmental stress, as microorganisms often rely on mutualistic relationships to enhance resilience (Cavaliere et al., 2017; Hernandez et al., 2021; Valiei et al., 2024). Additionally, it may suggest increased ecological niche overlap among species, particularly in resource-limited environments, where cooperation can be more advantageous than competition (Zhang et al., 2023). This is further supported by findings that mutualism and commensalism promote structural homogeneity and reduce variance in relative abundance, thereby conferring resilience and integrity to microbial communities (Peng et al., 2024). For the decrease in the average degree, it indicated a weakening of connectivity between microbial species and a concomitant deterioration in network structure. Furthermore, a decline in relative modularity may imply increased homogeneity of function among microbial communities, resulting in a less distinct functional division between different microbial species (Newman, 2006). This may, in turn, lead to a reduction in the communities’ adaptability to environmental changes (Sun, 2015). Collectively, these changes indicates that the conversion of black locust stands to coppice stands reduces the connectivity among fungal species, leading to a less organized network structure, increased functional homogeneity among microbial communities, diminished ecological functionality, and reduced resistance to environmental perturbations. Li et al. (2023) showed that microbial network complexity became more pronounced over the course of vegetation restoration in *R. pseudoacacia* plantations. Moreover, alterations in the interactions within the fungal community also reflect changes in the soil environment, suggesting that the soil habitat conditions for fungi may have deteriorated in conjunction with forest conversion.

In addition to the successional processes, seasonal changes can also lead to shifts in fungal communities and soil properties (Cao et al., 2021; Midgley and Phillips, 2019; Voříšková et al., 2014). Here, the seasonal dynamics of the soil fungal communities in the black locust stands showed time series characteristics as the distribution of the T001 group was steadily converged towards the T901 group (Supplementary Figure S1). For the differential species, no class differed between sample groups with similar temperatures, such as T901 vs. T001, and T904 vs. T910, suggesting that temperature does indeed affect fungal reproduction and growth, as well as modifying community structure to some extent (Supplementary Table S3). At the phylum level, Mucoromycota and Zoopagomycota differed in several pairs, with Mucoromycota being more abundant in the low temperature T901 and T001 groups than in the high temperature (T907), possibly due to their enhanced ability to withstand cold temperatures, as temperature selects for the survival of temperature-tolerant species and stimulates their activity in soil nutrient cycling (Guo et al., 2018; Melillo et al., 2002). Furthermore, a significant amount of research indicates that microbial respiration in the litter layer is higher in summer and autumn due to the optimal moisture and temperature conditions on the forest floor, which promotes increased microbial activity (Sato et al., 2019). In terms of the guilds, they exhibited significant differences across different seasons overall, and the temporal trends of the fungal community guilds in the FN and SN groups showed striking similarities, while the SN group showed notable differences over time (Figure 5). Although previous studies have identified temperature as a primary factor influencing fungal communities (Voříšková et al., 2014), no significant differences were observed between individual seasons, particularly between summer and winter. This lack of seasonal differentiation is likely attributable to the regulatory influence of other environmental factors, such as soil moisture and nutrient availability, which may buffer the effects of temperature on fungal communities. Upon targeted analysis of saprophytic and symbiotic fungi (Supplementary Table S5), we observed significant differences in their seasonal distributions. Saprophytic fungi were predominantly found in autumn and winter, whereas symbiotic fungi were more prevalent in spring, which can be illustrated bu. their ecological function in black locust forest. This pattern aligns with our third hypothesis and underscores the pivotal role of fungi in maintaining the stability and functionality of forest ecosystems.

Based on the network analysis (Supplementary Figure S4; Supplementary Table S6), we found that fungal cooperation was at its peak during the T904 period, likely due to the gradual emergence and growth of new leaves, resulting in increased water and nutrient uptake by roots. Forest soil fungi play a crucial role in supporting plant tree growth by assimilating essential nutrients (Bender et al., 2014; Lin et al., 2018; Treseder and Lennon, 2015). In T907, when soil temperature reached its peak for the year and was highly conducive to fungal growth, the complexity and the stability of the fungal community networks were strengthened, with the highest number of edges and average degree, but the lowest percentage of positive edges, indicating a decreased likelihood of cooperative relationships between fungi. For the network of the T910 group, both complexity and average degree decreased despite the abundance of litter present during this period. We hypothesize that the incomplete decomposition status of litter amplified the cooling effect of temperature reduction on soil fungal community dynamics. This proposition is supported by previous studies demonstrating that fresh autumn litter containing labile compounds undergoes accelerated decomposition during winter months (Snajdr et al., 2011). Notably, seasonal enzymatic analyses by Voříšková et al. (2014) revealed peak extracellular enzyme activity in winter across all soil horizons, particularly within the litter layer. In accordance with these biochemical observations, our network analysis detected enhanced fungal cooperation in winter-collected samples (T001), evidenced by a 4% increase in positive network edges. Furthermore, topological comparison revealed the highest similarity between fungal networks in T001 (winter) and T901 (autumn) groups. These findings not only align with established patterns of fungal community succession but also strengthen the empirical evidence supporting temperature as a key modulator of microbial community structure and interaction dynamics.

## 5 Conclusion

Our study shows that forest conversion from seedling to coppice stands, together with seasonal changes, significantly affects the composition of soil fungal communities, their guild structure and co-occurrence network patterns in black locust stands. Notably, the assembly patterns of fungal communities—rather than their richness and diversity—are primarily influenced by these factors. The observed shifts in dominant species, particularly the decrease in Ascomycota dominance and the increase in Basidiomycota, as well as changes in community stability and complexity, have important implications for soil health and ecosystem functionality. Specifically, the enhanced cooperative abilities among fungi in coppice stands to suggest potential benefits for soil fertility and plant growth, while the reduced complexity of co-occurrence networks may affect community resilience to disturbance. From a forest ecology and restoration perspective, our results highlight the need to intergrate soil fungal community dynamics into forest management strategies to ensure sustainable ecosystem health and functionality. Potential applications of our results include guiding forest restoration efforts and optimising management practices to enhance soil fungal diversity and interactions, thereby promoting ecosystem resilience and productivity.

## Data Availability

The original contributions presented in the study are included in the article/Supplementary material, further inquiries can be directed to the corresponding author.
