# Supplementary material for: Soil fungal community and co-occurrence network patterns at different successional stages of black locust coppice stands
Source: Front Microbiol. 2025 Mar 18;16:1528028. doi: 10.3389/fmicb.2025.1528028 (PMC11959006; doi:10.3389/fmicb.2025.1528028)
Supplement: Supplementary file 1 [file Data_Sheet_1.docx]

**Supplementary Materials**

**Fig. S1** Diversities of fungal communities in the FN, SN and TN groups. (A) Comparison of Chao1 and Simpson indexes of fungal communities. Horizontal bars in the box plots indicate median proportional values. Kruskal-Wallis test was applied to examine differences and different lower-case letters indicate significant differences (*p* < 0.05) among sampling groups. (B) PLS-DA plot comparison of fungal community profiles in the FN, SN and TN groups at the OTU level. (C) PLS-DA plot comparison of fungal community profiles along the timescale at the OTU level.

**Fig. S2** Distribution of fungal communities of each sample on a time scale at phylum level (A) and the class level (B). Class-level fungal community composition only showed the top 10 classes with the highest relative abundance in the bar plots.

**Fig. S3** ANOSIM analysis of the fungal community guilds in the growth scale (A) and time scale (B) based on Bray-Curtis distance

**Fig. S4** Co-occurrence network of the 400 most abundant OTUs in the T901, T904, T907, T910 and T001 sample groups (A-C). A connection stands for OTUs with Spearman correlation coefficient > 0.6 or < -0.6 and *p* < 0.05 were determined. The size of each node is proportional to the degree. The red edges indicate positive interactions between two OTUs, while blue edges indicate negative interactions. Colors of nodes in network represent the bacterial phyla.

**Fig. S5** Natural connectivity of co-occurrence network of T901, T904, T907, T910 and T001 groups.

**Table S1** The alpha indices of soil samples based on the high-throughput sequencing.

**Table S2** ANOSIM analysis results between two sample groups based on the Bray-Curtis distance. **Table S3** Difference analysis of fungi species across five sampling times at phylum and class level by STAMP at 95% confidence interval.

**Table S4** Guilds with significant difference across soil sample groups by STAMP confidence interval.

**Table S5** The relative abundance of symbiotic and saprotrophic fungi across soil sample groups (%).

**Table S6** Topological properties of co-occurrence networks for OTU composition of soil samples across different groups.


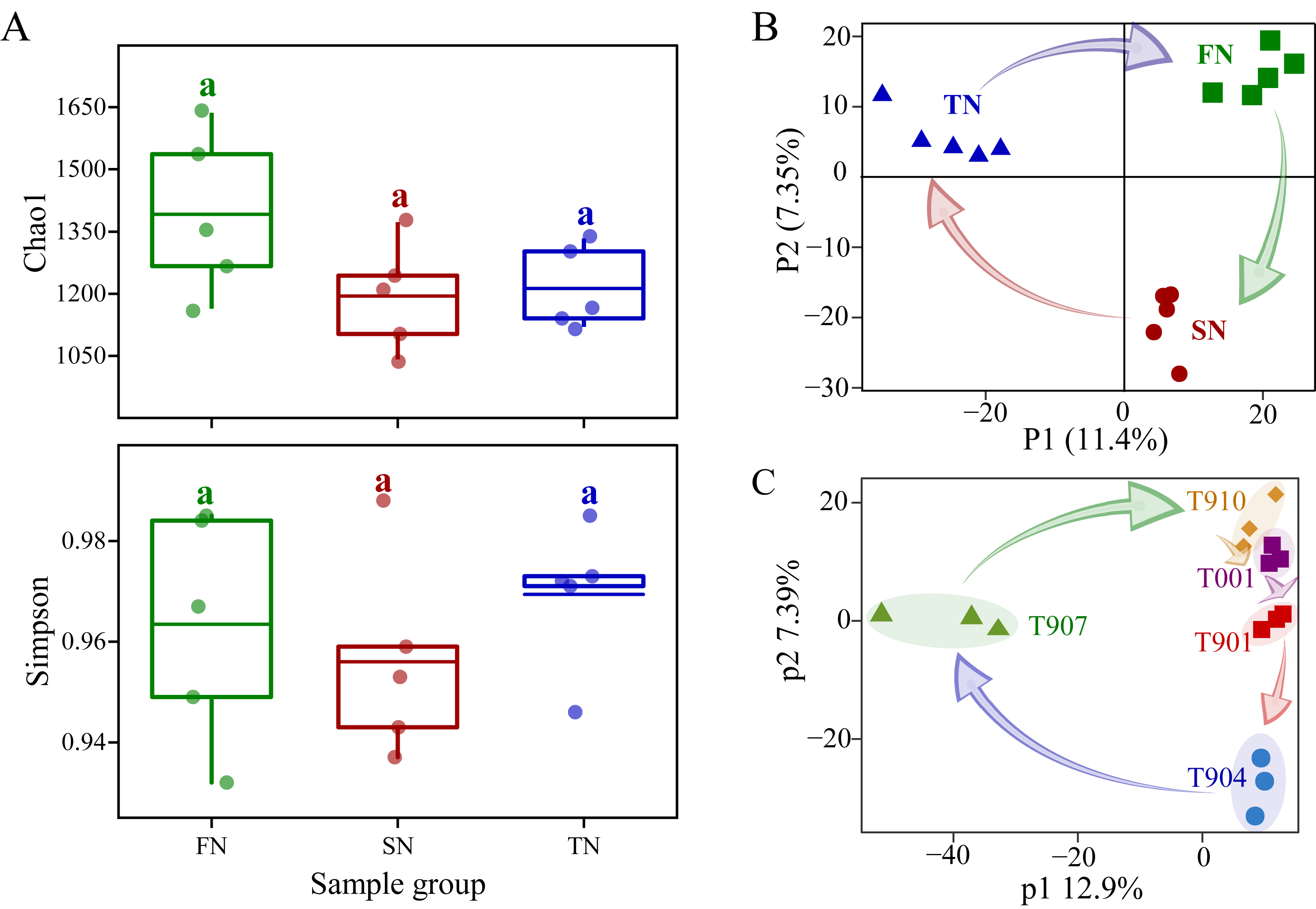


**Fig. S1** Diversities of fungal communities in the FN, SN and TN groups. (A) Comparison of Chao1 and Simpson indexes of fungal communities. Horizontal bars in the box plots indicate median proportional values. Kruskal-Wallis test was applied to examine differences and different lower-case letters indicate significant differences (*p* < 0.05) among sampling groups. (B) PLS-DA plot comparison of fungal community profiles in the FN, SN and TN groups at the OTU level. (C) (C) PLS-DA plot comparison of fungal community profiles along the timescale at the OTU level.


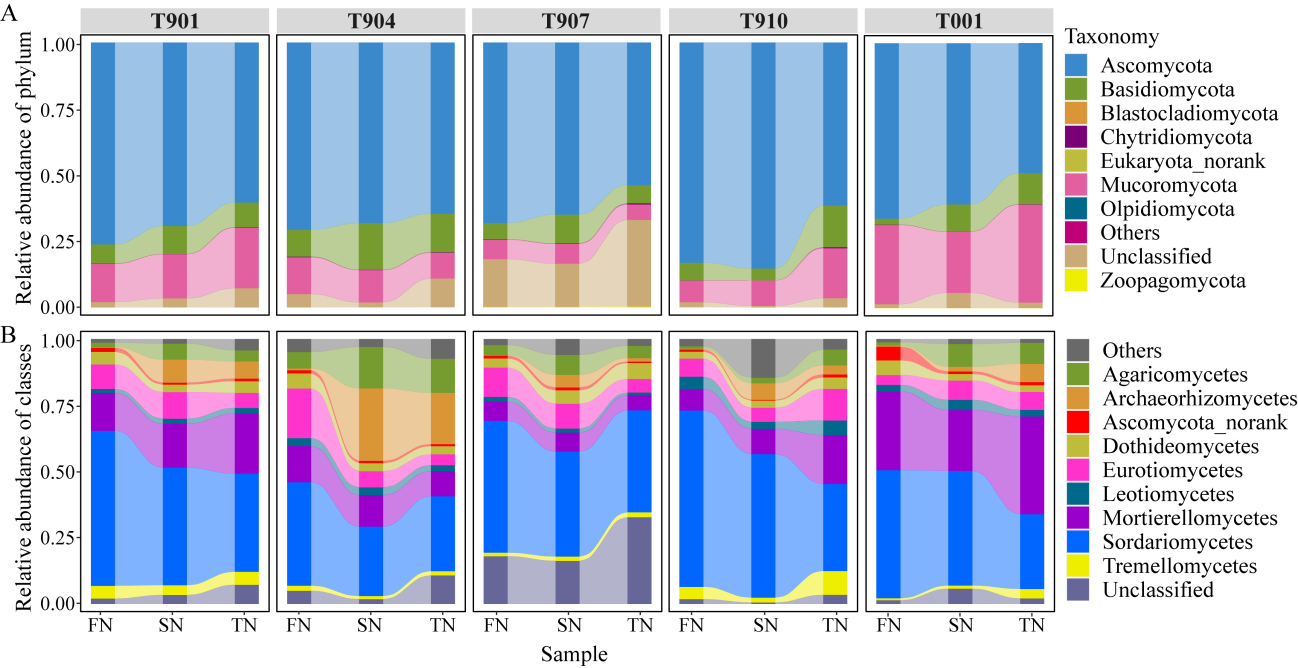


**Fig. S2** Distribution of fungal communities of each sample on a time scale at phylum level (A) and the class level (B). Class-level fungal community composition only showed the top 10 classes with the highest relative abundance in the bar plots.


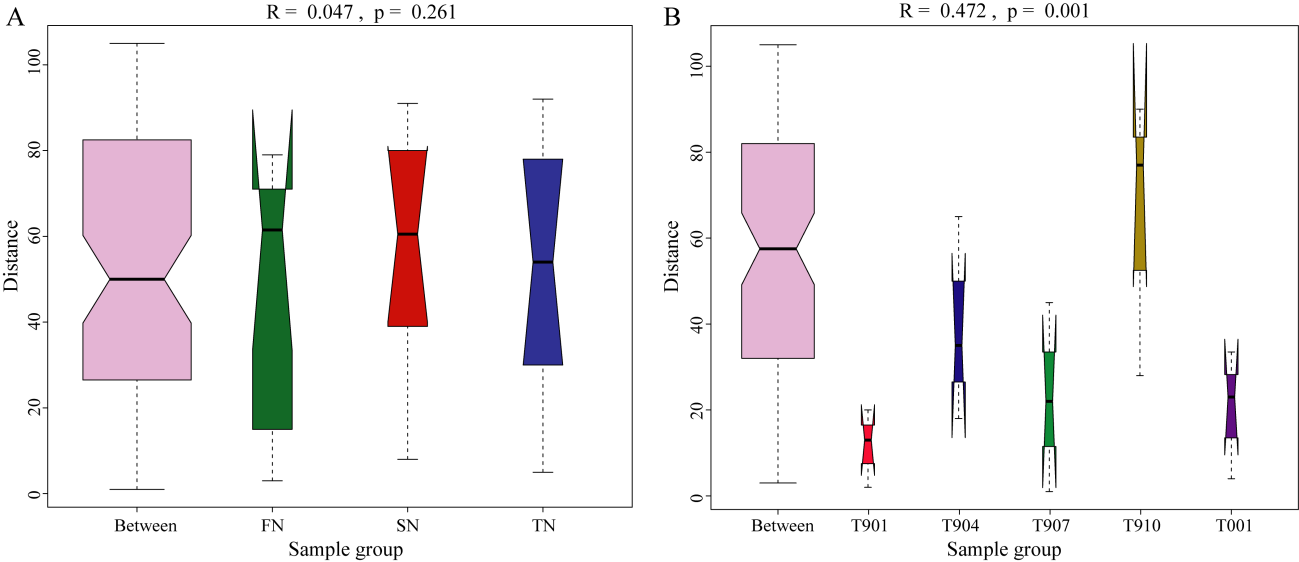


**Fig. S3** ANOSIM analysis of the fungal community guilds in the growth scale (A) and time scale (B) based on Bray-Curtis distance


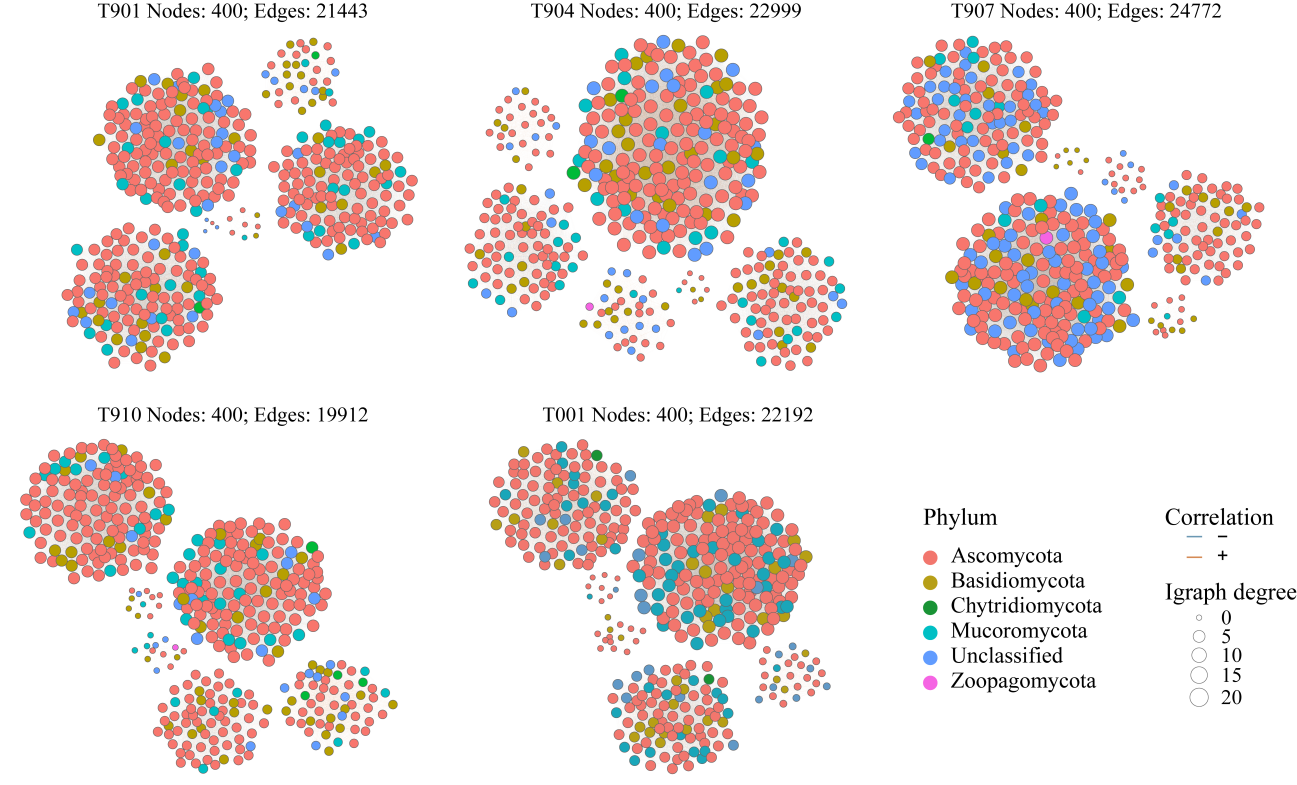


**Fig. S4** Co-occurrence network of the 400 most abundant OTUs in the T901, T904, T907, T910 and T001 sample groups (A-C). A connection stands for OTUs with Spearman correlation coefficient > 0.6 or < -0.6 and *p* < 0.05 were determined. The size of each node is proportional to the degree. The red edges indicate positive interactions between two OTUs, while blue edges indicate negative interactions. Colors of nodes in network represent the bacterial phyla.


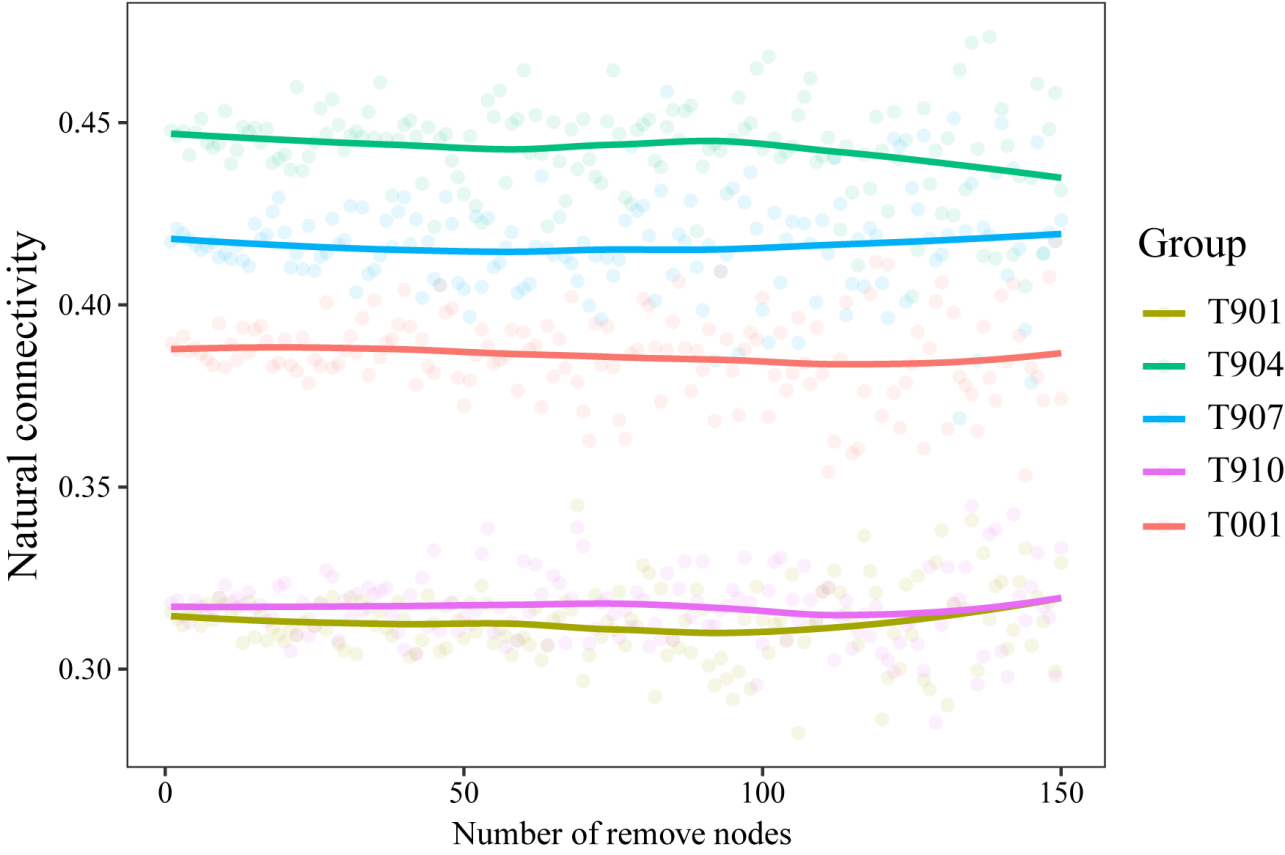


**Fig. S5** Natural connectivity of co-occurrence network of T901, T904, T907, T910 and T001 groups.

**Table S1** The alpha indices of soil samples based on the high-throughput sequencing.

| Sample | Group | | Richness | chao1 | Shannon | Simpson |
| --- | --- | --- | --- | --- | --- | --- |
| FN901 | FN | T901 | 1137 | 1537 | 4.552 | 0.967 |
| FN904 | FN | T904 | 1043 | 1354 | 5.224 | 0.985 |
| FN907 | FN | T907 | 1278 | 1642 | 5.22 | 0.984 |
| FN910 | FN | T910 | 917 | 1267 | 4.047 | 0.932 |
| FN001 | FN | T001 | 878 | 1159 | 4.102 | 0.949 |
| SN901 | SN | T901 | 884 | 1103 | 4.246 | 0.953 |
| SN904 | SN | T904 | 957 | 1244 | 4.123 | 0.937 |
| SN907 | SN | T907 | 1048 | 1210 | 5.356 | 0.988 |
| SN910 | SN | T910 | 823 | 1037 | 4.003 | 0.943 |
| SN001 | SN | T001 | 1023 | 1378 | 4.453 | 0.959 |
| TN901 | TN | T901 | 949 | 1302 | 4.628 | 0.971 |
| TN904 | TN | T904 | 932 | 1141 | 4.628 | 0.972 |
| TN907 | TN | T907 | 1153 | 1339 | 5.295 | 0.985 |
| TN910 | TN | T910 | 929 | 1166 | 4.702 | 0.973 |
| TN001 | TN | T001 | 833 | 1115 | 4.094 | 0.946 |

**Table S2** ANOSIM analysis results between two sample groups based on the Bray-Curtis distance.

| Comparison group | R | p value |
| --- | --- | --- |
| T901-T904 | 0.037 | 0.6 |
| T901-T907 | 0.852 | 0.1 |
| T901-T910 | 0.407 | 0.2 |
| T901-T001 | 0.259 | 0.2 |
| T904-T907 | 0.519 | 0.1 |
| T904-T910 | 0.111 | 0.4 |
| T904-T001 | 0.370 | 0.1 |
| T907-T910 | 0.667 | 0.1 |
| T907-T001 | 0.963 | 0.1 |
| T910-T001 | 0.407 | 0.2 |

**Table S3** Difference analysis of fungi species across five sampling times at phylum and class level by STAMP at 95% confidence interval based on Welch's t-test.

| Fungi | T901 vs  T904 | T901 vs  T907 | T901 vs  T910 | T904 vs  T907 | T904 vs  T001 | T907 vs  T910 | T907 vs  T001 | T910 vs  T001 |
| --- | --- | --- | --- | --- | --- | --- | --- | --- |
| Mucoromycota | ns | * | ns | * | * | ns | * | * |
| Zoopagomycota | ns | * | ns | * | ns | * | ** | ns |
| Number of differential phylum | 0 | 2 | 0 | 2 | 1 | 1 | 2 | 1 |
| Tremellomycetes | ** | * | ns | ns | ns | ns | ns | ns |
| Leotiomycetes | * | ns | ns | * | ns | ns | ns | ns |
| Mortierellomycetes | ns | * | ns | * | * | ns | * | * |
| Zoopagomycetes | ns | * | ns | * | ns | ns | * | ns |
| Orbiliomycetes | ns | ns | ** | ns | ns | ns | ns | * |
| Saccharomycetes | ns | ns | * | ns | * | ns | ns | * |
| Zoopagomycota  _norank | ns | ns | ns | ns | ns | * | ns | ns |
| Cystobasidiomycetes | ns | ns | ns | ns | ns | ns | * | ns |
| Malasseziomycetes | ns | ns | * | ns | ns | ns | ns | * |
| Number of differential classes | 2 | 3 | 3 | 3 | 2 | 1 | 3 | 4 |

**Table S4** Guilds with significant difference across soil sample groups by STAMP confidence interval.

| Guild | All sample^a^ | T901  vs  T904^b^ | T901  vs  T907^b^ | T901  vs  T910^b^ | T901  vs  T001^b^ | T904  vs  T907^b^ | T904  vs  T910^b^ | T904  vs  T001^b^ | T907  vs  T910^b^ | T907  vs  T001^b^ | T910  vs  T001^b^ |
| --- | --- | --- | --- | --- | --- | --- | --- | --- | --- | --- | --- |
| Endomycorrhizal-Plant Pathogen-Undefined Saprotroph | *** | * |  |  |  | * | * | * |  |  |  |
| Undefined Saprotroph | *** |  | * |  |  |  |  |  | * | * |  |
| Endophyte-Lichen Parasite-Plant Pathogen-Undefined Saprotroph | * |  | * | * | * |  |  |  | * | ** |  |
| Endophyte-Litter Saprotroph-Soil Saprotroph-Undefined Saprotroph | *** |  | * |  |  | * |  | * |  | * | * |
| Animal Pathogen-Undefined Saprotroph |  |  |  | * |  |  |  |  |  |  | * |
| Fungal Parasite-Plant Pathogen-Plant Saprotroph | * |  |  | * | *** |  |  |  |  |  |  |
| Wood Saprotroph | ** |  |  |  |  |  |  | * |  | * | * |
| Dung Saprotroph-Plant Saprotroph |  |  |  |  |  |  |  |  |  | * |  |
| Plant Pathogen |  |  |  |  |  |  |  |  |  | * |  |
| Undefined | * |  |  |  |  |  |  |  |  | * |  |

a, Krusksl-Wsllis H-test; b, Welch's t-test. * represents 0.01 < *p* < 0.05; ** represents 0.001 < *p* < 0.01; *** represents *p* < 0.0001

**Table S5** The relative abundance of symbiotic and saprotrophic fungi across soil sample groups (%).

| Sample | Symbiotic fungi | Saprotrophic fungi |
| --- | --- | --- |
| FN901 | 0.53 | 69.84 |
| FN904 | 2.52 | 69.48 |
| FN907 | 1.85 | 62.38 |
| FN910 | 1.51 | 74.20 |
| FN001 | 1.05 | 79.82 |
| SN901 | 0.69 | 64.42 |
| SN904 | 10.30 | 47.41 |
| SN907 | 1.87 | 54.11 |
| SN910 | 0.79 | 65.76 |
| SN001 | 2.72 | 74.65 |
| TN901 | 5.47 | 65.21 |
| TN904 | 8.98 | 48.09 |
| TN907 | 0.96 | 51.78 |
| TN910 | 1.52 | 68.34 |
| TN001 | 0.82 | 73.56 |
| FN | 1.49 | 71.14 |
| SN | 3.27 | 61.27 |
| TN | 3.55 | 61.39 |
| T901 | 2.23 | 66.49 |
| T904 | 7.27 | 54.99 |
| T907 | 1.56 | 56.09 |
| T910 | 1.27 | 69.43 |
| T001 | 1.53 | 76.01 |

The green and orange markings indicate the highest percentage of symbiotic and saprophytic fungi in the group

**Table S6** Topological properties of co-occurrence networks for OTU composition of soil samples across different groups.

| Properties | T901 | T904 | T907 | T910 | T001 |
| --- | --- | --- | --- | --- | --- |
| Number of nodes | 400 | 400 | 400 | 400 | 400 |
| Number of edges | 21443 | 22999 | 24772 | 19912 | 22192 |
| Percentage of positive edges | 0.521 | 0.618 | 0.505 | 0.542 | 0.536 |
| Percentage of negative edges | 0.479 | 0.382 | 0.495 | 0.458 | 0.464 |
| Edge density | 0.269 | 0.288 | 0.310 | 0.250 | 0.278 |
| Average degree | 107.215 | 114.995 | 123.86 | 99.56 | 110.96 |
| Number of clusters | 6 | 6 | 6 | 6 | 6 |
| Centralization degree | 0.057 | 0.168 | 0.118 | 0.079 | 0.120 |
| Relative modularity | 17.106 | 10.078 | 14.637 | 13.297 | 13.470 |
| Modularity random | 0.037 | 0.040 | 0.035 | 0.046 | 0.041 |
